# Bioactive Compounds for Skin Health: A Review

**DOI:** 10.3390/nu13010203

**Published:** 2021-01-12

**Authors:** Monika Michalak, Monika Pierzak, Beata Kręcisz, Edyta Suliga

**Affiliations:** 1 Department of Dermatology, Cosmetology and Aesthetic Surgery, The Institute of Medical Sciences, Medical College, Jan Kochanowski University, Żeromskiego 5, 25-369 Kielce, Poland; beata.krecisz@ujk.edu.pl; 2 Department of Nutrition and Dietetics, The Institute of Health Sciences, Medical College, Jan Kochanowski University, Żeromskiego 5, 25-369 Kielce, Poland; monika.pierzak@ujk.edu.pl (M.P.); edyta.suliga@ujk.edu.pl (E.S.)

**Keywords:** skin care, skin health, bioactive substances, phytonutrients, antioxidants, nutraceuticals

## Abstract

Human skin is continually changing. The condition of the skin largely depends on the individual’s overall state of health. A balanced diet plays an important role in the proper functioning of the human body, including the skin. The present study draws attention to bioactive substances, i.e., vitamins, minerals, fatty acids, polyphenols, and carotenoids, with a particular focus on their effects on the condition of the skin. The aim of the study was to review the literature on the effects of bioactive substances on skin parameters such as elasticity, firmness, wrinkles, senile dryness, hydration and color, and to define their role in the process of skin ageing.

## 1. Introduction

**Figure 1:**
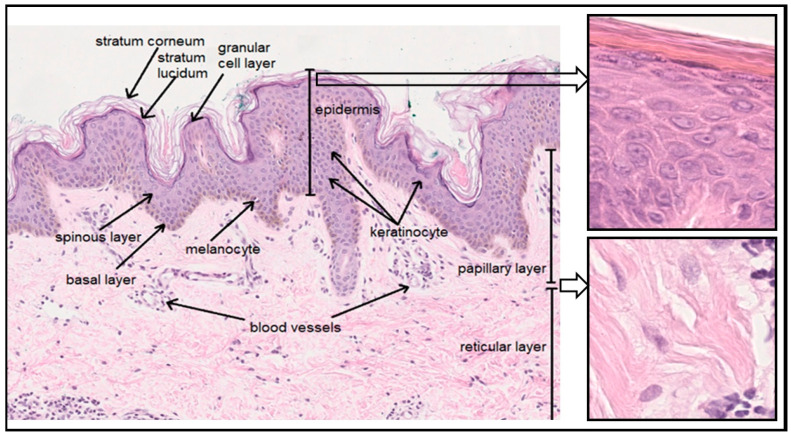
Skin structure (own work, photo: Department of Clinical and Experimental Pathology, Medical College, Jan Kochanowski University).

The skin is the largest organ of the human body. It is composed of the epidermis, which consists of epithelial tissue, and the dermis, which consists of connective tissue. Under the dermis, there is a layer of subcutaneous tissue called the hypodermis (Figure 1). The epidermis comprises a horny layer (stratum corneum), a clear layer (stratum lucidum), a granular layer (stratum granulosum), a spinous layer (stratum spinosum) and a basal layer (stratum basale). Apart from keratinocytes—cells involved in keratinization—the five-layer epidermis also contains pigment cells and melanocytes, as well as Langerhans cells, mastocytes, and Merkel cells. It is closely connected to the dermis underneath by the basement membrane. The dermis, which comprises a papillary layer (primarily loose connective tissue) and a reticular layer (dense connective tissue), contains fibroblasts responsible for the production of collagen, elastin, and glycosaminoglycans (GAGs), as well as numerous blood vessels, nerve endings, and appendages, such as hair follicles and sweat and sebaceous glands. The subcutaneous tissue consists of loose connective tissue containing fat cells (adipocytes) forming fat lobules [1,2,3].

The skin performs a wide variety of complex functions. It provides an optimal environment for deeper tissues by separating them from the external environment and, at the same time, ensures contact with it through the exchange of substances and reception of stimuli. The skin protects against biological agents (potentially pathogenic microbes), chemical agents (corrosive, irritating and allergenic substances) and physical factors (sunlight, ionizing radiation, infrared radiation, and mechanical and thermal factors). It performs important functions in water and electrolyte balance (epidermal barrier and sweat glands), thermoregulation (thermoreceptors) and the immune response (skin-associated lymphoid tissues (SALT)). It is also an important sensory organ (with free nerve endings, Pacinian corpuscles, Meissner corpuscles, Ruffini corpuscles, Krause end bulbs and Merkel cells). In addition, it is involved in metabolism and homeostasis and is responsible for the elimination, selective absorption and storage of substances [1,4]. 

One of the most common dermatological and cosmetic concerns is skin ageing: a natural, complex process influenced by two mechanisms—intrinsic (genetic, chronological) ageing resulting from the passage of time, and extrinsic ageing (photoaging), caused by environmental factors (including UV radiation, environmental pollution and cigarette smoke) [5,6,7,8]. The two processes overlap and are closely linked to increased reactive oxygen species (ROS) and oxidative stress in the skin [9]. Both the intrinsic and extrinsic processes are associated with biochemical disturbances (e.g., the excessive formation of oxygen radicals, leading to protein and DNA damage, amino acid racemization, and non-enzymatic glycosylation, leading to the abnormal cross-linking of collagen fibers and other structural proteins), as well as changes in the physical, morphological and physiological properties of the epidermis and dermis. These include disturbances in the function of the epidermal barrier, the flattening of the dermal–epidermal junction, a reduced number and activity of fibroblasts, the accumulation of abnormal elastin fibers (elastosis), and the impaired functioning of Langerhans cells [6,10,11,12,13]. Features characteristic of mature skin include wrinkles, a loss of elasticity, changes in color, uneven pigmentation and discoloring, dryness, foci of abnormal epidermal keratosis, telangiectasias, susceptibility to irritation, and slower skin regeneration and healing [5,8]. With age, the degradation of blood vessels leads to insufficient blood supply and, thus, the inadequate oxygenation and nourishment of the skin [8].

Many authors stress the relationship between a suitably balanced diet and the condition of the human body, including the appearance and functioning of the skin [14,15,16,17] (Table 1). The intake of essential nutrients in the daily diet is extremely important for the biological processes taking place in both young and ageing skin [15,18]. The skin is a tissue with high proliferative potential, which is why an adequate intake of proteins, carbohydrates and fats, which are essential for cellular generation, is so important [18,19,20]. The overall condition of the skin—its surface texture, color, and physiological properties—results from factors such as hydration, i.e., the presence of an adequate amount of water in the stratum corneum, sebum content and surface acidity. Natural moisturizing factor (NMF), consisting mainly of amino acids, plays an important role in hydration and acidity [15,21]. Specific fatty acids are also important for maintaining the function of the skin barrier and the integrity of the stratum corneum [22]. Research increasingly suggests that a well-balanced diet significantly affects the skin ageing process. Functional anti-ageing ingredients in food include substances involved in the synthesis and metabolism of skin components (e.g., protein peptides and essential fatty acids) and those that inhibit the degradation of skin components and maintain its structural integrity (e.g., substances regulating the expression of enzymes such as matrix metalloproteinases (MMPs) and activating protein 1 (AP-1)) [18]. Due to their ability to protect the skin from harmful UV-induced effects through their antimutagenic, antioxidant and free-radical-scavenging properties, some dietary botanicals could be useful supplements for mature skin care [23,24]. These include carotenoids and polyphenols, such as apigenin (a flavonoid occurring in numerous herbs, fruits and vegetables), quercetin (a flavonol found in onion skin and apple peel), curcumin (obtained from the turmeric rhizome), silymarin (a standardized extract of flavonolignans from milk thistle), genistein (an isoflavone from soybeans), proanthocyanidins (from the seeds of grapes), and resveratrol (a polyphenol found in grapes, peanuts, fruits, red wine and mulberries). Other key elements of an anti-ageing diet are vitamins and minerals with antioxidant properties [15,25].

The present study is a review of the literature on the effects of nutrition on the condition of the skin, as well as an attempt to establish whether a healthy diet supplemented with vitamins and minerals has photoprotective and anti-ageing effects. The aim of the study was to present the effects of bioactives on skin parameters such as a wrinkled appearance, elasticity, firmness, senile dryness, hydration, and color, as well as their role in the skin ageing process.

## 2. Selected Substances of Importance for Skin Function

The present study focused on substances that combat skin ageing and contribute to a younger-looking face with a healthy appearance; nutrients and dietary supplements reported to have the potential to prevent skin ageing are discussed. Particular focus is placed on substances that protect the skin against oxidative and UV-induced damage, dehydration and a loss of elasticity: vitamins A, C and E; selenium; zinc; copper; silicon; polyphenols; carotenoids; and essential polyunsaturated omega-3 and omega-6 fatty acids.

### 2.1. Vitamins

Vitamins are a group of compounds with diverse chemical structures that are essential for the normal functioning of the human body. They do not play an active role in supplying energy and are not building blocks for tissues, but they are essential for normal growth and development. They are biological catalysts and building blocks for the prosthetic groups of various enzymes, and thus, they enable numerous biochemical reactions at various levels [26].

#### 2.1.1. Vitamin A

**Figure 2:**
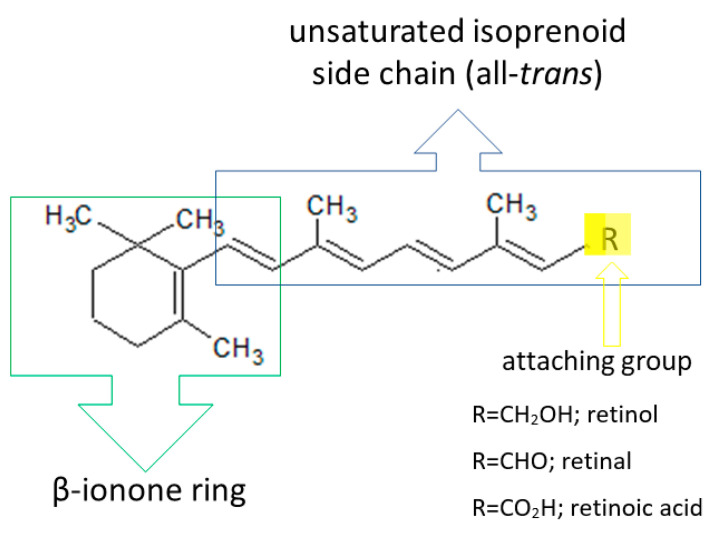
Basic structure of retinoids (own work based on [32]).

Vitamin A is one of the fat-soluble vitamins. The name refers to polyene compounds comprising a beta-ionone ring and a polyene side chain containing a functional group: an alcohol group, retinol; an aldehyde group, retinal; an acid group, retinoic acid; or an ester group, retinyl ester. Derivatives of vitamin A, called retinoids, include both natural and many synthetic retinol derivatives with activity similar to that of vitamin A [27,28,29,30,31] (Figure 2). 

**Scheme 1:**
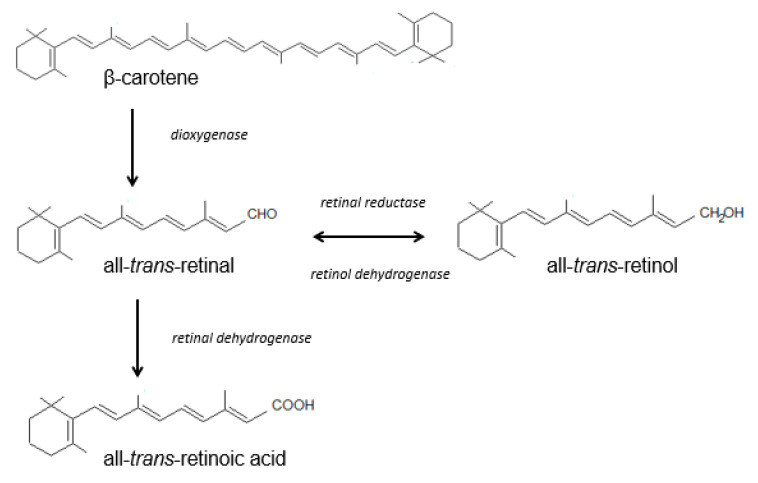
Metabolism of retinoids (own work based on [34,35]).

Vitamin A is obtained from the diet. Retinol and its various chemical forms are derived from foods of animal and plant origin (Table 1). Vitamin A from animal sources is mainly acquired in the form of retinyl esters (palmitate, propionate and acetate). Plants contain provitamin A (β-carotene), which is converted to vitamin A in the skin. Beta-carotene, one of the most important carotenoids, is a precursor of vitamin A in the human body [27,29,31,33,34] (Scheme 1). 

Beta-carotene is metabolized in the small intestine, leading to the formation of the aldehyde retinal, which can be reduced to retinol through enzymatic conversion. This process is reversible, and the alcohol can be converted as needed into retinal in an oxidation process catalyzed by retinol dehydrogenase. Further oxidation is irreversible and produces tretinoin. Tretinoin is thus the main metabolite of retinol, but it can be synthesized in certain tissues (e.g., the epidermal cells) without retinol, from retinal generated via the degradation of β-carotene. Retinyl esters in the enterocytes are converted to retinol, after which they are modified again and stored as retinyl esters in chylomicrons, before being released into the blood and supplied to cells and tissues associated with their carrier protein. The transport and activity of retinoids require specific proteins occurring in both the plasma (RBP—retinoid-binding proteins) and the cytoplasm (CRBP—cellular retinoid-binding proteins). In cell nuclei, retinoic acid receptors (RARs) and retinoid X receptors (RXRs, for all retinoids) have been detected [27,29,31,33,34]. In the blood and tissues, the two dominant endogenous retinoids are retinol and its esters. Retinol and retinyl esters account for more than 99% of all the retinoids present in the skin [36].

Vitamin A and all of its derivatives, applied systemically or topically, have a significant influence on skin health. Retinoids are a group of chemical compounds that inhibit cell division during excessive proliferation and activate it when the process is too slow. They play an active role in protein production, cellular metabolism and cell division [28,34,37]. Retinoids also affect the thickness and color of the skin, regulate the function of the sebaceous glands, and limit sebum production, as well as being responsible for hair and nail growth, and also influencing the distribution of melanin in the skin [27,30,35]. 

The anti-ageing effects of retinoids are mediated by three major types of skin cells: epidermal keratinocytes, dermal endothelial cells and fibroblasts [38]. Topically applied retinoids penetrate the keratinized epidermis and, to a lesser extent, the dermis and subcutaneous tissue [27,30,35]. A study in mice by Törmä et al. showed that retinol esters accounted for 90% of the vitamin A in the epidermis, with retinol making up the remaining 10% [39]. After penetrating the epidermis, retinoids play a role in the release of transcription and growth factors. They are responsible for the renewal of the epidermis through their active role in the exfoliation of dead cells in the stratum corneum and the proliferation of live cells in the epidermal layers [28,34,35]. Moreover, vitamin A promotes the compaction of the stratum corneum and deposition of GAGs in the stratum corneum and intercellular spaces in the epidermis [40]. A study conducted by Kang et al. showed that the application of all-trans-retinol on normal human skin induced epidermal thickening and increased the mRNA expression of cellular retinoic acid binding protein and cellular retinol binding protein [41]. Retinoids also reduce transepidermal water loss (TEWL) by strengthening the epidermal barrier [28,34,35]. After penetrating the dermis, retinoids increase the synthesis of elastin and collagen by stimulating the fibroblasts [42]. Topical retinol improves the dermal extracellular matrix (ECM) microenvironment (type I collagen, fibronectin, and elastin) and activates ECM-producing cells in aged skin [32,38]. The activation of fibroblast production is stimulated through the TGF-β/CTGF pathway, the major regulator of ECM homeostasis [38]. Retinoids and all biologically active forms of vitamin A also promote the remodeling of reticulin fibers and synthesis of a new capillary network in the dermis. This process influences the condition of the connective tissue of the dermis, improving its firmness, hydration and elasticity [35,42]. Retinoids protect collagen against the destruction induced by MMPs, as well as enhancing the synthesis of tissue inhibitors of metalloproteinases (TIMPs) [32,42]. Varani et al. observed that topical vitamin A (retinol) reduced MMP expression and increased fibroblast growth and collagen synthesis in naturally aged, as it did in photoaged, skin [42]. Pierard-Franchimont et al. showed that a retinol formulation improved the physical properties of ageing facial skin, including tensile properties and contours, after 12 weeks of treatment [43].

Retinol and retinyl esters exhibit a strong capacity to absorb ultraviolet radiation in the range of 300–350 nm [39]. Antille et al. conducted a study to assess the ability of retinyl palmitate to protect the skin, prevent DNA damage and protect against the induction of erythema by a single exposure to strong UVB radiation. The results clearly indicate that retinyl esters present in the epidermis have strong antiphotocarcinogenic properties and protect DNA against damage by UV radiation [44]. 

Topically applied retinoids reduce the discoloration of the skin and its pigmentation by about 60%. Retinoids influence the function of melanocytes and the distribution of melanin in the skin, as well as blocking the transport of melanin to epidermal cells. A decrease in the number of melanocytes is related to the inhibition of melanogenesis [32].

Retinoids have been shown to improve the clinical features of aged appearance by a reduction in fine wrinkling, increased smoothness and decreased hyperpigmentation [45]. Vitamin A and its biologically active forms, retinoids, are used in the care of ageing skin and to eliminate signs of photoaging, but also in the treatment of numerous dermatological conditions, which may be seborrheic (e.g., acne vulgaris and rosacea), viral (flat warts, molluscum contagiosum or genital warts induced by the human papilloma virus (HPV)), proliferative, cancerous or precancerous (keratoacanthoma, cutaneous T-cell lymphoma, leukoplakia of the oral mucosa, actinic keratosis and xeroderma pigmentosum), autoimmune (lupus or lichen sclerosus), or papulosquamous dermatoses (psoriasis, pityriasis rubra pilaris, or lichen planus), as well as genodermatoses with keratosis disorders (congenital and hereditary ichthyosis or Darier disease) [27,28,44].

#### 2.1.2. Vitamin C

**Figure 3:**
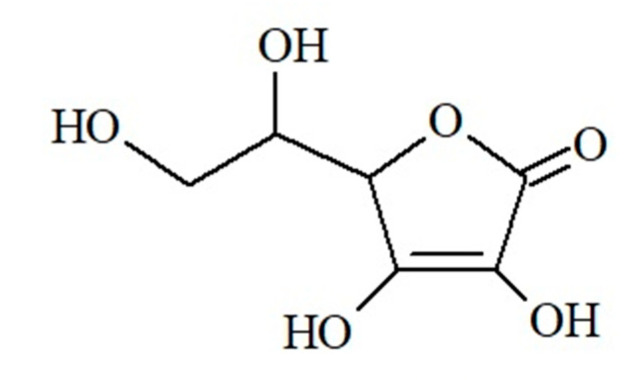
Chemical structure of ascorbic acid (own work).

Vitamin C (L-ascorbic acid) is a highly water-soluble, sugar-like alpha-ketolactone produced from D-glucose (Figure 3). 

As humans lack L-gulonolactone oxidase, the enzyme enabling its production, this vitamin must be supplied in the diet (Table 1). About 70–80% of it is absorbed in the duodenum and the proximal part of the small intestine. The absorption and bioavailability of vitamin C are affected by the current metabolic state of the body, as well as by age and sex. Digestive and absorption disorders, vomiting, diarrhea, smoking, and certain medicines, such as aspirin, significantly affect its concentration in the body [46,47,48]. Reserves of vitamin C are stored in organs with high metabolic activity, such as the liver, pancreas, lungs, brain and adrenal glands [49]. The vitamin C concentration in the skin is higher than in other human tissues, at 6–64 mg/100 g wet weight in the epidermis and 3–13 mg/100 g in the dermis [50,51,52]. There are two transport mechanisms for ascorbic acid, and these depend on the sodium–ascorbate cotransporters (SVCTs) present in various tissues and organs. In the skin, sodium–ascorbate cotransporter-1 (SVCT1) is responsible for the transport of epidermal vitamin C to the keratinocytes, while sodium–ascorbate cotransporter-2 (SVCT2) is responsible for intradermal transport [53,54].

Vitamin C promotes the formation of the epidermal barrier and collagen in the dermis, protects against skin oxidation, helps to counteract skin ageing, and plays a role in the signaling pathways of cell growth and differentiation, which are linked to the occurrence of various skin diseases [55,56].

The role of vitamin C in differentiating keratinocytes is being researched. The vitamin has been shown to enhance keratinocyte differentiation, reduce differentiation-dependent oxidative stress and maintain the integrity of the skin barrier, which in turn helps to prevent water loss from the skin [55,57]. Pasonen-Seppanen et al. showed that vitamin C enhanced the differentiation of rat epidermal keratinocytes cell line in an organotypic culture model, resulting in a stronger stratum corneum, which indicates that it has a skin-protective function [58]. Savini et al., in an in vitro study on normal human epidermal keratinocytes, showed that ascorbate improves the barrier functions of the epidermis and promotes the formation of the cornified cell envelope, a specialized structure that protects the body against harmful chemical and physical factors [57]. Research by Cosgrove et al. indicates that a higher vitamin C intake is associated with a reduced likelihood of a wrinkled appearance and senile dryness [59]. 

The biological function of vitamin C in the skin is its active role in collagen synthesis. It is responsible for the biosynthesis of collagen through its role in the hydroxylation of proline and lysine residues to hydroxyproline and hydroxylysine. It donates electrons to enzymes involved in hydroxylation, resulting in the conversion of procollagen to collagen [46,49]. It functions as a cofactor of proline and lysine hydroxylases, which are responsible for the tertiary structure of collagen; promotes the expression of collagen genes; and activates the production of collagen mRNA by fibroblasts [50]. Vitamin C exhibits anti-wrinkle activity. There is scientific evidence that ascorbic acid plays an active role in the proliferation and migration of skin fibroblasts and stimulates the production of collagen and elastin in cultures of fibroblasts [46,49]. Supplementation with vitamin C enhances the production of GAGs, promotes the expression of genes coding for antioxidant enzymes and enzymes involved in DNA repair, and inhibits the production of pro-inflammatory cytokines and apoptosis induced by UV radiation or other harmful environmental factors [49].

The antioxidant properties of vitamin C protect the skin, especially the epidermis, against oxidants generated by ultraviolet radiation and other environmental factors. Although the direct antioxidant protection provided by vitamin C is limited to aqueous compartments, vitamin C significantly curtails lipid oxidation by regenerating fat-soluble vitamin E; it regenerates α-tocopherol from α-tocopherol radicals found in cell membranes and lipoproteins [60]. Topical vitamin C supplementation can also counteract UVA-induced oxidative stress [61]; Offord et al. assessed the photoprotective potential of vitamin C and other antioxidants, confirming that vitamin C plays a significant role in protecting the skin against UVA radiation [62]. The photoprotective efficacy of vitamin C is enhanced by combining it with vitamin E [63]. Placzek et al., in a study with human volunteers, showed that the oral administration of ascorbic acid and D-alpha-tocopherol for three months significantly reduced the sunburn reaction to UVB irradiation. The results of that study also suggest that supplementation with a combination of the two antioxidants protects against DNA damage [64]. Eberlein-König and Ring have also shown that vitamin C used in combination with vitamin E and, to a lesser extent, with other photoprotective compounds dramatically increases their photoprotective effects as compared to monotherapies [65].

Vitamin C is also thought to be involved in inhibiting melanogenesis. Melanin, a natural skin pigment resulting from complex biochemical conversions in melanocytes catalysed by tyrosinase, plays an important role in photoprotection. The overproduction of melanin, however, may contribute to hyperpigmented diseases as well as the initiation of melanomas [66,67]. A review of the literature indicates that vitamin C inhibits melanogenesis with low cytotoxicity, although some studies suggest that the role of vitamin C in inhibiting melanogenesis is very minor [67,68,69]. Furthermore, a combination of vitamin C and other vitamins, including vitamin E, is more effective at reducing melanin than vitamin C alone and can be used for treating skin discoloration, e.g., age spots or melasma [50,68].

Numerous scientific papers emphasize the potential of vitamin C for the treatment of such diseases as porphyria cutanea tarda, atopic dermatitis, malignant melanoma, herpes zoster and postherpetic neuralgia, as well as the clinical application of this vitamin in the treatment of other skin conditions, such as acne, acne scars, allergic contact dermatitis, psoriatic progressive pigmented purpuric dermatosis (PPPD), genital herpes and vitiligo [56,70,71,72,73,74,75].

#### 2.1.3. Vitamin E

**Figure 4:**
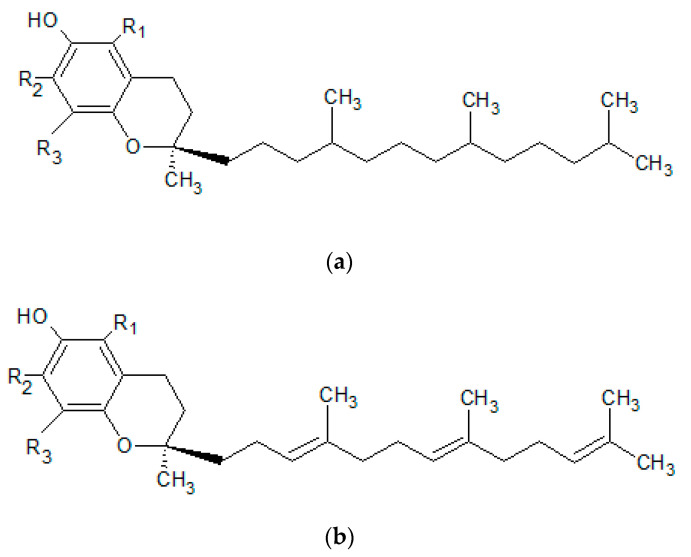
Basic structure of (**a**) tocopherols and (**b**) tocotrienols (own work).

Vitamin E is a group of lipophilic compounds that includes four tocopherols (α-, β-, γ- and δ-tocopherol) and four tocotrienols (α-, β-, γ- and δ-tocotrienol) [76,77]. The chemical structures of all the compounds referred to as vitamin E have a two-ring 6-hydroxychroman skeleton and a side chain composed of three isoprenoid units. Tocopherols have a chromanol ring and a saturated phytyl tail, while the chemical structures of tocotrienols have an unsaturated tail [76,77,78] (Figure 4). 

Vitamin E is exclusively synthesized by plants [76], and all forms are supplied to the human body by food (Table 1). Alpha-tocopherol is the most important form, showing an affinity for the specialized protein alpha-TTP, which binds and transports this form of the vitamin only. The remaining dietary forms are metabolized in the liver and eliminated from the body with bile [79,80]. 

Vitamin E plays an important role in maintaining skin health, and it has been used for over 50 years in dermatology. The most probable physiologic function of epidermal vitamin E is contributing to the antioxidant defences of the skin and protecting the epidermis and dermis against oxidative stress induced by environmental factors. Vitamin E is the major lipid-soluble antioxidant in humans [40]. Owing to the antioxidant properties of vitamin E and its ability to scavenge free radicals and become part of lipid structures, it protects against lipid peroxidation and slows skin ageing [80]. Alpha-tocopherol has been shown to decrease the amount of 8-hydroxydeoxyguanosine produced indirectly by reactive oxygen species, so it may reduce ROS-induced DNA damage and thereby help to retard the development of skin cancer [81].

Alpha-tocopherol supplementation has been shown to improve facial hyperpigmentation. Ichihashi et al., in an in vitro study using cultured human melanoma cells and normal human melanocytes, showed that alpha-tocopherol inhibits tyrosine hydroxylase activity and suppresses melanogenesis [81]. Thus, vitamin E may be a candidate whitening agent for the treatment of hyperpigmentation, including age-related conditions or those arising from sun exposure. 

Some research also indicates that vitamin E displays strong photoprotective, firming, hydrating and anti-ageing properties, as well as improving the elasticity, structure and softness of the epidermis and dermis [40,82]. It is thought that vitamin E, incorporated into the intercellular cement and lipid structures, protects the skin against solar UVB radiation and, thus, against redness and swelling. Although the topical application of alpha-tocopherol shows promising photoprotective effects, especially when combined with systemic and topical antioxidant substances such as vitamin C or carotenoids, controlled studies in humans are needed before vitamin E can be recommended as an effective cosmeceutical anti-ageing agent [40,83].

The available literature concerning the efficacy of the systemic and topical use of vitamin E is extensive, but the results are often contradictory and range from an improvement in skin appearance to no effect at all [40]. Vitamin E plays a role in the healing of wounds of varying aetiology; in the treatment of dermatological conditions such as subcorneal pustular dermatoses, cutaneous amyloidosis, atopic dermatitis, epidermolysis bullosa, psoriasis, acne vulgaris and scleroderma; in skin cancer prevention; and in the treatment of Hailey–Hailey disease [82]. Tsoureli-Nikita et al. conducted a study to evaluate the effect of oral vitamin E supplementation on the improvement and remission of atopic dermatitis. The results, which indicated relationships between vitamin E, the IgE level and the clinical symptoms of atopy, suggest that vitamin E can be used to treat atopic dermatitis [84]. Butt et al. evaluated the usefulness of vitamin E in protecting skin against thermal trauma. The results suggest that the clinical transplantation of keratinocytes preconditioned with vitamin E, alone or in combination with skin fibroblasts in skin substitutes, could be used to treat thermally damaged skin [85]. There are conditions for which vitamin E has no confirmed effects, including keratosis follicularis, chronic cutaneous lupus erythematosus, pseudoxanthoma elasticum, and porphyria cutanea tarda [82]. Other studies have also failed to confirm that the topical application of vitamin E improves the cosmetic appearance of scars [86]. 

### 2.2. Minerals

Minerals, alongside vitamins, are essential micronutrients that cannot be synthesized in humans and therefore must be obtained via the diet. A healthy diet should ensure an adequate intake of both macroelements (calcium, phosphorus, potassium, sodium and magnesium) and microelements (iodine, sulphur, zinc, iron, chlorine, cobalt, copper, manganese, molybdenum and selenium). Minerals are responsible for the functioning of the skeletal, circulatory, nervous and endocrine systems. They also have numerous health benefits as cofactors and coenzymes in various enzyme systems, aiding the regulation and coordination of biochemical and physiological functions. A deficiency of microelements has an adverse effect on human development and health, including the functioning and appearance of the skin [26,87]. In the context of skin ageing, attention is also paid to the role of minerals such as selenium, zinc, copper and silicon.

#### 2.2.1. Selenium

Selenium (Se) is one of the most important trace elements without which the body cannot function properly. In terms of its chemical structure, selenium is one of the chalcogens. It occurs in two forms: inorganic (selenates and selenites) and organic (selenomethionine and selenocysteine) [88]. Selenium should be supplied to the human body with foods of plant and animal origin [26,88] (Table 1).

Selenium plays a role as part of the structure of enzymatic proteins. It is also an element of three families of enzymes: glutathione peroxidases (GPx), thioredoxin reductases (TrxR) and iodothyronionic deionidases (DIOs). Twelve single selenoproteins occur in the human body, including selenoprotein P, responsible for controlling redox potential in cells and the transport of selenium to peripheral tissues. Selenium exhibits strong antioxidant properties and protects against DNA damage [88,89]. 

The mineral is also important for skin function, having a protective effect and the ability to scavenge free radicals [89]. By stimulating the activity of selenium-dependent antioxidant enzymes, such as glutathione peroxidase and thioredoxin reductase, selenium protects the skin against the oxidative stress induced by UV radiation [87]. Zhu et al. draw attention to the role of selenium in protecting skin cells against the ageing induced by UVB radiation [90]. This was confirmed in a study by Jobeili et al., which showed that selenium delays skin ageing by protecting keratinocyte stem cells [91]. Favrot et al. showed that low doses of Se (30 nM) provide potent protection against UVA-induced cytotoxicity in young keratinocytes (from 20–30-year-old donors), while higher concentrations (240 nM) were required for protective efficacy in old keratinocytes (from donors 60–70 years old) [92]. 

Selenium supplementation is an important strategy for inhibiting wrinkles, because it can reverse ultraviolet light damage and play an anti-ageing role in the skin [89,92]. Kim et al. showed that a selenium-rich tuna heart extract enhanced collagen synthesis and promoted the proliferation of skin fibroblasts, thus having anti-ageing and anti-wrinkle effects [93]. These studies indicate that Se supplementation could be a new strategy for combating skin ageing and photoaging. 

Due to the anti-inflammatory and antioxidant properties of selenium, some research has focused on its role in skin cancer prevention. Whether selenium can prevent skin cancer or not is controversial. Research by Pols et al. indicates that a high serum concentration of selenium is associated with an approximately 60% decrease in the incidence of basal cell carcinoma (BCC) and squamous cell carcinoma (SCC) of the skin [94]. However, some researchers have demonstrated that supplementation with Se does not significantly affect the incidence of BCC or is ineffective in preventing it [95,96]. 

#### 2.2.2. Zinc

Zinc (Zn) is an essential microelement with an important role in many physiological processes [97]. A deficiency can cause a number of health problems, so it is essential to ensure an adequate intake, mainly through a balanced diet containing both animal and plant products, but also by addressing factors that can impede the absorption of this element. Zinc can be supplied in food products (Table 1) or in the form of dietary supplements [26,97,98]. The element has been shown to be present in all human tissues and body fluids. The average content of this element in an adult is 2–3 g [97,99]. Zinc is present in the muscles, bones, skin and liver, as well as in the brain, kidneys, spleen and pancreas [97,99,100,101]. The skin is the third most Zn-abundant tissue in the body, with greater amounts in the epidermis than in the dermis [99,102]. In the epidermis, zinc is more abundant in the stratum spinosum than in the other layers of keratinocytes, while in the dermis, the zinc concentration is higher in the upper parts than in the deeper areas [102,103]. 

Zinc performs catalytic, structural and regulatory functions [97,98], being important for the regulation of lipid, protein and nucleic acid metabolism, as well as gene transcription [98]. The molecular mechanisms of action of zinc are linked to its involvement in the structure and function of over 300 enzymes, including oxidoreductases, transferases, hydrolases, lyases, isomerases and ligases [97,104]. Zinc is also a component of numerous DNA transcription factors (zinc fingers) [100,105]. 

Zinc ions are involved in maintaining the balance between oxidation and reduction. As an antioxidant, zinc protects the cells of the body against the harmful effects of free radicals. An adequate level of this element is required for the activity of antioxidant enzymes, including cooper zinc superoxide dismutase (Cu/Zn-SOD), which is responsible for neutralizing the superoxide radical [97]. 

Zn is essential for the division and differentiation of new cells, and it plays a role in apoptosis and the ageing of the body [99,100]. The element influences the structure and proper functioning of the skin and mucous membranes [98,100]. It stabilizes skin cell membranes and participates in basal cell mitosis and differentiation [106], while also playing crucial roles in the survival of keratinocytes. The ZIP2 protein, a zinc transporter, is essential for the differentiation of keratinocytes [103].

Zinc also affects the immune function of the skin, modulating macrophage and neutrophil functions, phagocytic activity and various inflammatory cytokines [98,99]. It plays an important role in preventing the damage induced by UV radiation, influences collagen metabolism, exhibits antiandrogen properties by modulating the activity of type 1 and 2 5*α*-reductase, and also promotes lipogenesis and glucose transport through insulin-like effects on 3T3-L1 fibroblasts and adipocytes [98,107,108]. The oral and/or topical administration of Zn has long been used for its effect on skin regeneration and healing [99,109]. Topical preparations such as calamine or zinc pyrithione have been used as soothing agents or active ingredients in anti-dandruff shampoos [98]. Zinc preparations in the form of pastes or ointments cleanse the skin of excess sebum, restore its natural pH, and have astringent, anti-inflammatory and anti-acne effects. Zinc oxide, due to its ability to reflect and disperse UV rays, is used as a physical filter in sunscreens [97,98,99,107,110]. 

Zinc is used to treat numerous dermatological conditions, such as infections (e.g., warts), inflammatory dermatoses (acne vulgaris, rosacea, atopic dermatitis and alopecia areata) and pigmentary disorders (melasma) [97,98,99,107,110]. Zinc administered orally or topically has been shown to have therapeutic applications in skin ageing (a 0.1% copper–zinc malonate cream applied topically for 6 weeks significantly reduced wrinkles) [111]; melasma (a 10% zinc sulphate solution applied topically twice daily for 2 months significantly reduced MASI scores) [112]; actinic keratoses (a 25% zinc sulphate solution applied topically twice daily for 12 weeks was safe and effective, especially in patients with multiple actinic keratosis lesions) [113]; xeroderma pigmentosum (a 20% topical application of a zinc sulphate solution for 4 months to 2 years improved all types of skin lesions, softened the skin, lightened the skin color, and cleared the skin of solar keratosis and small malignancies) [114]; eczema (a 0.05% Clobetasol + 2.5% zinc sulphate cream applied topically was effective in hand eczemas) [115]; rosacea (100 mg of oral zinc sulphate three times per day was effective after 3 months of therapy) [116]; and alopecia areata (5 mg/kg/day, in three divided doses, of oral zinc sulphate induced significant hair growth after 6 months) [117].

#### 2.2.3. Copper

Copper (Cu) is naturally found in many food sources [26,118] (Table 1). Dietary cupric copper (Cu^2+^) is reduced by several reductases to cuprous copper (Cu^+^) and taken up by copper transporter 1 (CTR1) at the plasma membrane [119]. In the human body, copper has been found in the bones, muscles, skin, bone marrow, liver and brain [120]. More than 30 proteins essential for living organisms (e.g., lysyl oxidase, dopamine β-hydroxylase, cytochrome *c* oxidase, superoxide dismutase and tyrosinase) contain copper [121], so Cu is involved in numerous physiological and metabolic processes important for the functioning of the human body [122].

Copper also exhibits a wide range of antimicrobial activity, including that against Gram-positive (e.g., *Staphylococcus aureus*, *Enterococcus faecalis*, *Clostridium difficile* and *Listeria monocytogenes*) and Gram-negative (e.g., *Escherichia coli*, *Pseudomonas aeruginosa* and *Klebsiella pneumoniae*) bacteria, fungi (*Candida albicans* and *Aspergillus brasiliensis*) and viruses *(adenovirus*, *norovirus* and *poliovirus*) [123,124,125]. Copper and copper compounds have been used for centuries by many civilizations for general hygiene and to treat various conditions, such as headaches, burns, intestinal worms, and ear infections, as well as skin diseases [118,126]. Pulverized malachite (basic cupric carbonate) was used by the Sumerians for generic medical purposes and by the ancient Egyptians to prevent and cure eye infections. The ancient Chinese and ancient Romans also used various copper compounds to treat eye and skin diseases [118]. Preparations containing copper have been used throughout history in cases of chronic inflammation, skin eczemas, tuberculosis, syphilis and lupus [126]. Copper sulphate is widely used in Africa to heal wounds. Copper ions reduce the risk of the fungal and bacterial infection of minor wounds and cuts, as well as enhancing wound healing [118]. 

Copper is also important for the overall condition of the skin. Cu stimulates the proliferation of dermal fibroblasts and is involved in the synthesis and stabilization of extracellular matrix skin proteins and angiogenesis. It is a cofactor of superoxide dismutase (SOD), an enzyme involved in protecting the skin against the harmful effects of free radicals, and also prevents oxidative damage to cell membranes and lipid peroxidation [118,126]. Copper deficiency reduces the activity of the enzyme Cu/Zn-SOD and ceruloplasmin, as well as copper-independent enzymes, including catalase and glutathione peroxidase. Copper also promotes the function of free radical scavengers such as metallothionein and glutathione. A deficiency of copper ions in the plasma, liver, erythrocytes and heart increases the concentration of the lipid peroxidation product malondialdehyde (MDA) [127].

Furthermore, Cu is a cofactor of tyrosinase, the main enzyme involved in synthesis of the skin pigment melanin [128]. In the active center of the enzyme, in the N-terminal domain, there are two copper atoms, which are coordinated by three histidine residues. Three types of tyrosinase are involved in melanogenesis: oxytyrosinase (E_oxy_), mettyrosinase (E_met_) and deoxytyrosinase (E_deoxy_) [129]. 

Copper ions are commonly included in cosmetics. Cu is used as an active component of face creams, including those for the care of mature, tired, dull or oily skin [118,130]. The effectiveness of the active ingredients is known to depend on their ability to penetrate the skin [130,131]. A study by Mazurowka and Mojski showed that the peptides glycyl-histydyl-lysine (GHK) and γ-glutamyl-cysteinyl-glycine (GSH) influence the permeation of copper ions through the horny layer of the epidermis [131]. The copper tripeptide complex (GHK-Cu and GSH-Cu) plays an important role in the protection and regeneration of skin tissue, increases the synthesis of collagen and elastin, and improves the condition of ageing skin. For this reason, copper peptides are often used as cosmetic ingredients [130]. In comparison with other metal compounds, copper has a low allergenic potential, and the risk of adverse side effects due to skin contact with the metal is small [132,133].

#### 2.2.4. Silicon

Silicon (Si) is the second most abundant element on Earth, present in water, plants and animals [134] and it has semi-metallic properties. In nature, it is not normally found in free form but is usually present as a chemical compound of silicon dioxide, a complex compound, or silicon silicate [135,136]. The silicon present in food is solubilized in the acidic environment of the stomach, becoming easily absorbed orthosilicic acid (OSA) [134]. Si is mainly absorbed from the diet [135,137] (Table 1). Another important source of silicon used in dietary supplements is horsetail (*Equisetum arvense*) [134]. 

Silicon is present in all healthy tissues of the human body, including the connective tissues, bones, liver, heart, muscle, kidneys and lungs [137]. Si is also present in the skin, hair and nails [138]. The amount of silicon in tissues decreases with age, most likely because the organ responsible for silicon absorption is the thymus, which atrophies with age [137]. 

Organic silicon plays an important role in skin structure, promotes neocollagenesis, strengthens connective tissue, and reduces the risk of alopecia [139]. Orthosilicic acid (OSA) stimulates fibroblasts to secrete collagen type I [140]. Si also promotes the synthesis of elastin and GAGs, helps to preserve blood vessel elasticity, and increases the resistance and thickness of nail and hair fibers [135]. Barel et al. demonstrated that the oral intake of choline-stabilized orthosilicic acid for 20 weeks had a significant positive effect on the surface and mechanical properties of skin. Treatment with silicon may also improve keratin structure in the hair and nails and reduce brittleness [138]. Supplementation with silicon in a highly bioavailable form can be used for skin rejuvenation [141]. Kalil et al. described the positive effect of orthosilicic acid stabilized with hydrolyzed marine collagen at 600 mg/day on skin texture, firmness and hydration [141]. Ferreira et al. investigated the oral intake of two forms of Si—maltodextrin-stabilized orthosilicic acid (M-OSA) and monomethylsilanetriol (MMST)—and their effects on the nails, skin and hair. They were found to improve skin parameters, increase eyelash length, reduce facial wrinkles and UV spots, and act as aluminum detoxification agents [135]. Barel et al. studied the effect of supplements containing choline-stabilized orthosilicic acid (ch-OSA) on the skin, hair and nails. In a group of 50 women with photodamaged facial skin taking 10 mg of Si/day orally for 20 weeks, significant improvements were observed in the surface characteristics and mechanical properties of the skin, as was a positive effect on the brittleness of the hair and nails [138].

Silicones are included in cosmetic formulations such as hair conditioners and shampoos to give hair a silky appearance and shine, as well as in facial creams to protect and improve the skin’s strength and elasticity [134,136]. Silicone products applied to the skin impart a pleasant smoothing sensation. Silicone elastomeric particles can absorb various liquids, including emollients and sebum, and can thus be used as an agent to carry active ingredients to the skin or to control sebum deposition in the skin [136,142].

### 2.3. Fatty Acids

**Figure 5:**
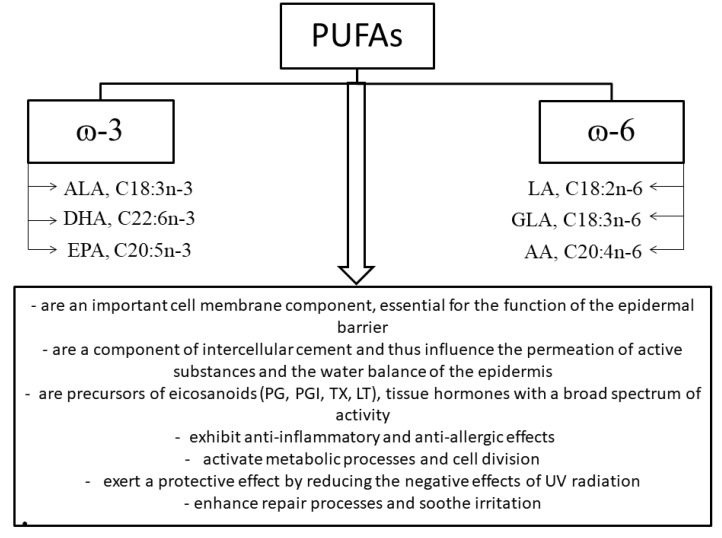
Classification of polyunsaturated fatty acids and the effects of omega-3 and omega-6 fatty acids on physiological processes in the skin; ALA, alpha-linolenic acid; DHA, docosahexaenoic acid; EPA, eicosapentaenoic acid; LA, linoleic acid; GLA, gamma-linolenic acid; AA, arachidonic acid; PG, prostaglandins; PGI, prostacyclins; TXA, thromboxanes; LT, leukotrienes (own work based on [151,153,154]).

Unsaturated fatty acids include omega-9 (ω-9; n-9) monounsaturated fatty acids and omega-3 (ω-3; n-3) and omega-6 (ω-6; n-6) polyunsaturated fatty acids (PUFAs) [151]. Omega-3 and omega-6 fatty acids, including alpha-linolenic (ALA), linoleic (LA) and gamma-linolenic (GLA) acids, included among the essential fatty acids (EFAs), are of significant dietary and cosmetic importance (Figure 5) [152].

Essential fatty acids are ascribed an important role in prophylaxis, especially of cardiovascular disease and allergic or inflammatory conditions [155]. EFAs also play an important role in skin structure and function. 

Owing to unsaturated fatty acids, which, alongside ceramides and cholesterol, are components of the intracellular cement, the skin can act as an effective barrier limiting TEWL, thus ensuring adequate hydration and protecting against external factors [153,154,156]. EFAs have therapeutic properties (e.g., anti-inflammatory and anti-allergic) and exert protective effects. The results of recent research on the beneficial effects of GLA on various dermatological conditions are promising and support the hypothesis that it is an essential fatty acid for skin function. GLA applied topically as a cream penetrates to the stratum corneum, while taken orally, it reaches the dermis, enhancing its cohesiveness and preventing excessive TEWL [156,157].

The symptoms of fatty acid deficiency include dryness of the epidermis, peeling, flabby skin, skin inflammation, an increased susceptibility to irritation and slower healing. A deficiency of LA, a component of ceramide 1, which plays an important role in the cohesiveness of the intracellular cement, results in the dysfunction of the skin barrier and symptoms of dry skin. Research by Cosgrove et al. showed that a higher LA intake is associated with a lower likelihood of senile dryness and skin atrophy [59]. Many skin problems, including excessive exfoliation of the epidermis, are also caused by a deficiency of GLA, which is formed from LA via an enzymatic reaction involving delta-6 desaturase [153,154,158,159]. A deficiency of essential fatty acids can also reduce the fluidity of sebum, which leads to the obstruction of the sebaceous glands and the appearance of blackheads and inflammation [152,157].

Unsaturated fatty acids are not synthesized in the human body, so they must be supplied through the diet. An important source of EFAs is vegetable oils obtained from seeds, fruit, nuts and sprouts [156]. The biological value and cosmetic suitability of oils depend on their percentages of fatty acids, both saturated (e.g., stearic and palmitic acid) and unsaturated (e.g., oleic, linoleic, linolenic and arachidonic acid). Vegetable oils rich in LA include wheat germ, soybean, sunflower seed and sesame seed oil [152,160], while oils from the seeds of borage, evening primrose or blackcurrant are important sources of GLA [161,162]. Oils rich in EFAs improve skin hydration, have a regenerative effect on the damaged epidermal lipid barrier, and regulate skin metabolism. Vegetable oils play an important role in the care of dry, sensitive, oily, acne-prone and mature skin [152,157] (Table 2).

Omega-3 acids obtained from fish oil—eicosapentaenoic acid (EPA) and docosahexaenoic acid (DHA)—also play an important role in skin function. Although these acids are not present in the normal epidermis, their metabolites (epidermal 15-lipoxygenase transforms EPA into 15-hydroxyeicosapentaenoic acid (15-HEPE) and DHA into 17-hydroxydocosahexaenoic acid (17-HDoHE)) accumulate in it after the consumption of fish oil [185]. There are studies indicating that fish oils have a protective role and can reduce the severity of erythema. Rhodes et al., in a study with a group of 15 people given fish oil containing 1.8 g of EPA and 1.2 g of DHA, observed a marked reduction in UVB-erythemal sensitivity after six months of supplementation [186]. In a randomized double-blind trial in 42 healthy patients taking 4 g/day of EPA or monounsaturated oleic acid for three months, an increase in the UV-induced erythematous threshold was observed, as was a decrease in the expression of the protein p53, a marker of DNA damage induced by UV radiation. The results indicate that EPAs have a UV-filtering effect and suggest that longer-term supplementation may reduce the risk of skin cancer in humans [187]. Furthermore, the supplementation of diets with vegetable or fish oils may generate local cutaneous anti-inflammatory metabolites, which could serve as adjuncts in the management of skin inflammatory disorders [185].

### 2.4. Polyphenols

**Figure 6:**
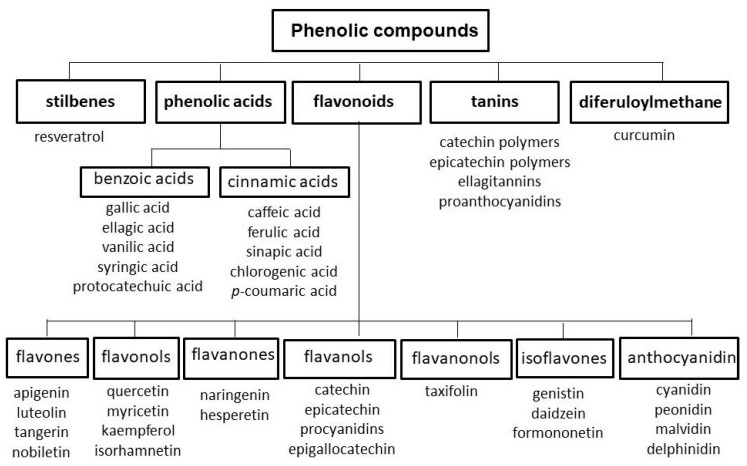
Classification of polyphenols (own work based on [190,191,192,193]).

Polyphenols are compounds widespread in the world of plants, imparting color—ranging from red to yellow to blue—to flowers and fruits [188]. Rich sources of polyphenols include spices and herbs, such as cloves (eugenol), star anise (anethole), peppermint (eriocitrin), oregano (pinocembrin), sage, thyme, spearmint and rosemary (rosmarinic acid), as well as fruits, including berries (black chokeberries, black elderberries, blueberries and blackcurrants), plums, cherries, strawberries, raspberries, grapes and drupe fruits (apples, peaches, apricots, nectarines and pears), some seeds (flaxseeds and soybeans), nuts (chestnuts, walnuts, hazelnuts, pecans and almonds) and vegetables, including black and green olives, artichoke heads, red and green chicory, onions, spinach, broccoli, asparagus and lettuce [189]. Polyphenols are organic chemical compounds containing two or more hydroxyl groups attached to an aromatic ring [190]. Five main groups of phenolic compounds can be distinguished based on chemical structure: flavonoids, phenolic acids, tannins, stilbenes and diferuloylmethane [190,191,192] (Figure 6). 

**Figure 7:**
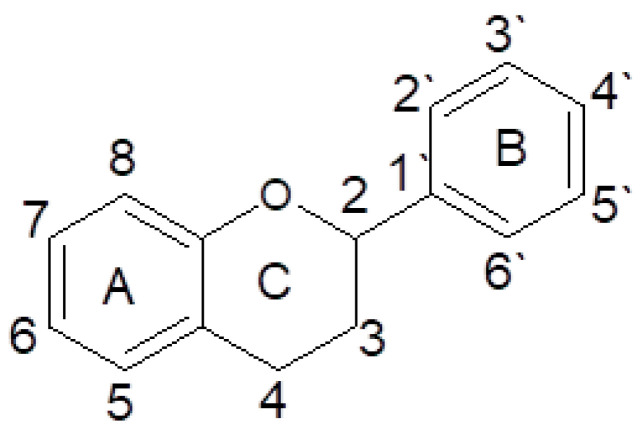
Basic structure and numbering system of flavonoids (own work).

The most thoroughly researched group of polyphenols is the flavonoids [193]. The structure of flavonoids is based on a flavan structure consisting of three rings [193,194] (Figure 7). 

The health-promoting effects of polyphenols taken orally are linked to their bioavailability, which is largely dependent on their chemical structure. The bioavailability depends on the amounts of nutrients that are digested, absorbed and included in metabolic processes [195,196]. The diversity of polyphenols’ structures is reflected in their multi-faceted biological activity [190]. They exhibit anti-inflammatory, antibacterial, antifungal, antiviral, antiallergic, anticancer and anticoagulant properties [190,191,192,193,197]. Plant polyphenols are considered important substances for skin function, with hydrating, smoothing, softening, soothing and astringent effects [25,190,193,197,198,199]. Polyphenols inhibit the activity of enzymes present in the skin—collagenase and elastase, which catalyze the hydrolysis of collagen and elastin fibers, and hyaluronidase, which degrades hyaluronic acid. Furthermore, they soothe irritation and reduce the redness of skin, accelerating the natural regeneration of the epidermis, stabilizing the capillaries, improving microcirculation and elasticity in the skin, and protecting against harmful external factors, including UV radiation [188,197,198,200]. 

The presence of antioxidants has been shown to be linked to a lower frequency of ROS-induced photoaging [201]. The antioxidant and antiradical properties of polyphenols result from the elimination of radicals through direct reactions, scavenging, or the reduction of free radicals (e.g., hydroxyl, superoxide, peroxide and alcoxyl radicals) to less reactive compounds. Polyphenols can also chelate transition metal cations (e.g., Cu^2+^ and Fe^2+^), thus preventing Haber–Weiss and Fenton reactions (which lead to the generation of the extremely reactive hydroxyl radical •OH) and also inhibiting the activity of many enzymes involved in free radical generation (e.g., xanthine oxidase, protein kinase and lipoxygenase). Their activity also involves the stimulation and protection of other antioxidants, such as ascorbate in the cytosol or tocopherol in biological membranes [194,195,202]. 

Raw polyphenolic materials exert synergistic effects with other antioxidants in preventing skin ageing. Cho et al. investigated the effect of the oral administration of an antioxidant mixture of pycnogenol, evening primrose oil, vitamin C and vitamin E on UVB-induced wrinkle formation. The study showed that a 10-week administration of the antioxidants to hairless mice irradiated three times a week with UVB radiation significantly prevented the UVB-induced expression of MMPs and mitogen-activated protein (MAP) kinase, as well as the activation of the transcription factor AP-1. It also enhanced the expression of type I procollagen and TGF-β2). The results indicate that the oral administration of the antioxidant mixture can inhibit wrinkle formation by preventing the expression of MMPs and increasing collagen synthesis [203].

Examples of polyphenolic plant materials of value in the prevention of skin ageing and protection of skin cells against UV radiation and photoaging, present in the diet or used as ingredients in dietary supplements, include sylimarin, a complex of the flavonolignans silibinin, isosilibin, silychristin and sylidianin obtained from milk thistle seed coats (*Silybum marianum*) [204]; genistein, an isoflavonoid obtained from soybeans (*Glycine max*) [205]; curcumin, a component of the spice turmeric (*Curcuma longa*) [206]; and resveratrol, a polyphenolic phytoalexin present in grape seeds (*Vitis vinifera*) [207]. 

Another source of polyphenols (anthocyanins and hydrolysed tannins) with beneficial effects on the condition of the skin is pomegranates (*Punica granatum*) [208]. Research has confirmed the photochemoprotective, antioxidant, anti-inflammatory and antiproliferative properties of pomegranate extract. Pomegranate fruit extract has been shown to reduce UVB-induced oxidative stress and the oxidation of skin proteins [209], and also to improve the color of the skin and restore the glow of skin exposed to UV radiation [210]. 

Another material rich in polyphenols exhibiting properties that protect skin cells is green tea. The leaves of *Camelia sinensis* contain four major polyphenols: epicatechin (EC), epicatechin gallate (ECG), epigallocatechin (EGC) and epigallocatechin gallate (EGCG), the last of which accounts for 40% of all the polyphenols [211]. Green tea polyphenols (GTPs) are an example of a component of plant-based dietary supplements used to prevent the adverse biological effects of UV radiation, including immunosuppression and photocarcinogenesis [212]. Research also indicates that GTPs can alleviate the symptoms of premature skin ageing induced by UVB radiation [213]. Chiu et al. studied the effect of GTPs on the symptoms of photoaging of the skin in a double-blind trial using a placebo in a group of 40 women. In half of the subjects a 10-percent green tea cream was applied together with 300 mg, twice-daily green tea oral supplementation, while the other half received the placebo. Histological examination showed significant improvements in elastic tissue content, but no significant changes were demonstrated clinically. The authors concluded that a clinically perceptible improvement might require a longer period of GTP administration. GTPs also exhibit anti-inflammatory and antioxidant properties and the ability to scavenge free radicals [214]. In vivo studies indicate an increase (from 2 to 15%) in plasma antioxidant activity following the consumption of tea or tea polyphenols [215]. A study by Vayalil et al. demonstrated the inhibition of oxidative stress induced by UVB radiation in the skin of SKH-1 hairless mice that received GTPs in their drinking water (0.2%, *w*/*v*) and were then exposed to multiple doses of UVB light (90 mJ/cm^2^) for two months. The inhibition of protein oxidation was observed, as was the inhibition of the UVB-induced expression of matrix-degrading MMPs, such as MMP-2 (67%), MMP-3 (63%), MMP-7 (62%) and MMP-9 (60%) [213]. A study in C57BL/6 mice showed that green tea extract reduced collagen cross-linking and UVB-induced oxidative changes in the structures of proteins [216]. 

A particularly interesting substance of plant origin that can function as a functional analogue of retinol is bakuchiol. This is a phenolic compound with a monoterpene side chain, obtained from the seeds and leaves of *Psoralea corylifolia*. Chaudhuri and Bojanowski showed that bakuchiol can prevent wrinkles; improve pigmentation, elasticity and firmness; and reduce overall photodamage. The authors stress that this compound can be used as a retinol-like anti-ageing functional compound [37].

The literature includes reports of many clinical trials evaluating the effectiveness of polyphenol-based therapies. Polyphenols used topically and orally may be effective in treating certain dermatological conditions, including anogenital warts, alopecia, acne vulgaris, fungal infections, hyperpigmentation and photoaged skin [217].

### 2.5. Carotenoids

**Figure 8:**
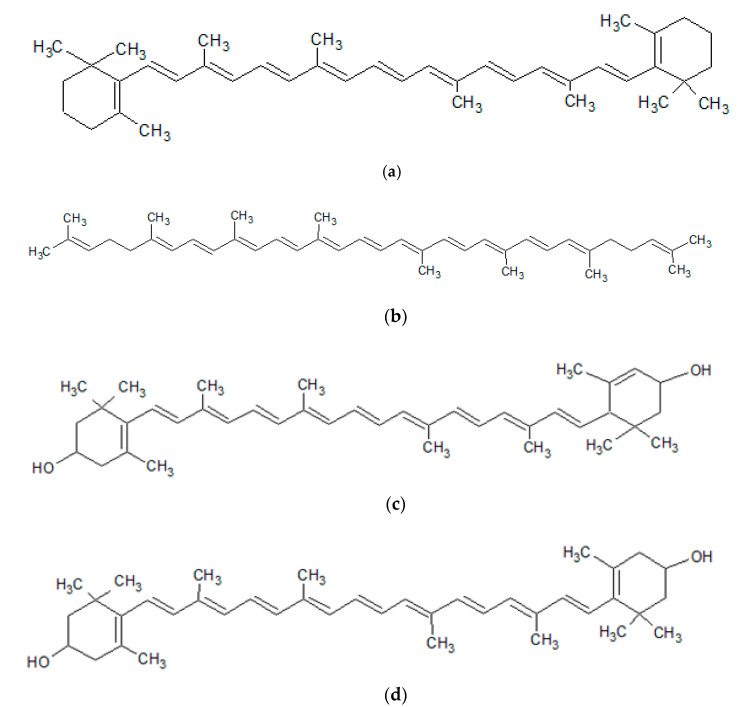
Structures of the major carotenoids: (**a**) β-carotene; (**b**) lycopene; (**c**) lutein; (**d**) zeaxanthin (own work).

Carotenoids are polyene pigments, having a conjugated system of double bonds. They can occur in the form of acyclic, monocyclic or bicyclic compounds. They include carotenes and their oxygenated derivatives, xanthophylls [218,219,220] (Figure 8). 

Carotenoids are present in plants—imparting a yellow-to-red color to the flowers, fruits or leaves—and in the marine environment, as a red–orange pigment common in many aquatic animals [220,221,222] (Table 3).

Among 800 recognized carotenoids, about 40 are present in the typical human diet, while only 14 have been identified in the blood and tissues [220,224]. In the human body, carotenoids mainly accumulate in lipid tissue cells and the liver. They are also present in the horny layer of the epidermis, as confirmed by Raman spectroscopy. The content of carotenoids in human skin varies, with the highest levels observed in parts of the body with high concentrations of sweat and sebaceous glands (e.g., the forehead and hands). The total content of carotenoids in the skin is influenced by numerous factors, such as the intake of fruits and vegetables, their bioavailability from various foods, supplementation, exposure to UV radiation, air pollution, alcohol consumption, smoking and stress [220,229]. The external application of preparations containing carotenoids in combination with oral supplementation has been shown to increase the concentrations of these compounds in the skin [25,230]. 

Due to the coloring properties of carotenoids, they are often used in the food, pharmaceutical, cosmetics and feed industries [220,225]. 

Carotenoids prevent ageing, stimulate fibroblasts to produce collagen and elastin, inhibit the activity of MMPs, and exhibit anti-inflammatory and UV-filtering effects [222,226,231]. They have been shown to improve the elasticity, hydration and texture of skin; reduce TEWL; lighten the skin; reduce discoloration; and delay the signs of photoaging [222,231,232,233]. Yoon et al. investigated the effect of astaxanthin (2 mg/day) and collagen hydrolysate (3 g/day) supplementation on moderately photoaged skin in humans. The bioactives significantly improved barrier integrity, reduced TEWL, and increased the expression of procollagen type I mRNA. The results indicate that astaxanthin combined with collagen hydrolysate, due to the beneficial effects of the compounds on skin elasticity and hydration, can be used as an anti-ageing agent for photoaged skin [234]. A study by Juturu et al. showed that supplementation with 10 mg of lutein and 2 mg of zeaxanthin isomers daily for 12 weeks improves the appearance of skin and lightens skin tone [233]. Schwartz et al. evaluated the effect of zeaxanthin on skin parameters such as fine and deep lines, total wrinkles, wrinkle severity, radiance/skin color, discoloration and skin pigment homogeneity. The results indicate that a zeaxanthin-based dietary supplement and topical serum improve hydration and reduce wrinkles in facial skin [235]. Furthermore, carotenoids support healing processes and protect the skin against the oxidative stress resulting from excessive ROS activity, thereby reducing the risk of skin cancer [220,226,232,236,237]. Research confirms the photoprotective effects of carotenoids in the diet and in the form of dietary supplements. Wertz et al. investigated this effect in the case of β-carotene. This compound was found to suppress the UVA induction of MMP-1, MMP-3 and MMP-10, the three main metalloproteinases involved in photoaging. Moreover, β-carotene was shown to block the ^1^O_2_-mediated induction of MMP-1 and MMP-10 in a dose-dependent manner [228]. Cesarini et al., in a group of 25 subjects, studied the effect of an orally administered antioxidant complex consisting of β-carotene, lycopene, selenium and α-tocopherol on parameters of the protection of the epidermis against UV-induced skin damage. After 6 weeks of the application of the antioxidants, an increase in protection against UV-induced skin damage was observed, accompanied by a reduction in lipoperoxide levels and sunburn cells (SBCs), as well as an increase in skin pigmentation [238]. Other research has shown a significant reduction in UV-induced erythema and improvement in the hydration and elasticity of skin following the oral application of lutein [237]. Heinrich et al. compared the erythema-protective effect of 24 mg/d of beta-carotene from an algal source to that of 24 mg/d of a carotenoid mixture (beta-carotene, lutein and lycopene, at 8 mg/d each). An increase in carotenoid levels in the serum and skin was observed after 12 weeks of supplementation in both groups, as was a comparable reduction in UV-induced erythema [239]. Aust et al. evaluated the photoprotective effects of synthetic lycopene in comparison with those of a tomato extract and a drink containing solubilized tomato extract. The subjects ingested similar amounts of lycopene (about 10 mg/d) from all three sources. After 12 weeks of supplementation, significant increases in serum lycopene levels and total skin carotenoids were observed in all the groups, as was less severe erythema following exposure to UV radiation. The protective effects were more pronounced in the groups that received the tomato extract or a drink containing solubilized tomato extract [240]. These studies indicate that a photoprotective effect and reduction in the severity of UV-induced erythema can be ascribed to the entire family of carotenoids. 

Researchers draw attention to role of carotenoids in the prevention and treatment of photodermatoses, such as erythropoietic protoporphyria (EPP), porphyria cutanea tarda (PCT) and polymorphous light eruption (PMLE) [220]. Some studies indicate that a high dietary intake of lutein and zeaxanthin is associated with a reduced incidence of squamous cell carcinomas (SCC) in subjects with a history of skin cancer at baseline [241]. In other research, treatment with β-carotene did not significantly reduce the occurrence of new skin cancers in persons with previous non-melanoma skin cancer [242].

## 3. Conclusions

The skin is a sensitive indicator of nutritional deficiencies. The most effective way to improve the condition of the skin is to supply it with essential nutrients, both externally and—importantly—internally, through a varied diet. An increasing body of research suggests that a well-balanced diet significantly affects the skin ageing process. It is worth noting the substances that protect and restore the epidermal barrier, which reduces TEWL, ensuring an appropriate level of skin hydration and protecting against external factors and the damage induced by inflammation (e.g., omega-3 and omega-6 fatty acids). Antioxidants and other phytonutrients that scavenge ROS and alleviate oxidative skin damage also play an important role in the prophylaxis and care of ageing skin, as do substances that protect the skin against the negative effects of ultraviolet radiation (including vitamins A, C and E; selenium; zinc; copper; silicon; polyphenols; and carotenoids). The oral administration of antioxidants can be an effective supplement to chemical and physical UV-filtering agents and can reduce the DNA damage leading to skin ageing and the development of skin cancer. The inclusion of these substances in the daily diet could be a useful approach in anti-ageing interventions. In conclusion, the promotion of healthy dietary habits can benefit the appearance of the skin, delay ageing processes and reduce the risk of skin cancer. 

## Figures and Tables

**Table 1:** Sources and roles of vitamins and minerals of importance for skin health and function.

Micronutrient	Source	Functions in Skin	Hypovitaminosis and Skin Disorders	References
Vitamin A (retinol)	liver, fish liver oils, dairy products, butter, cheese, egg yolk, meat products, certain saltwater fish, plant products (β-carotene)	-stimulates synthesis of epidermal proteins -stimulates proliferation of epidermal cells and regulates exfoliation of keratinocytes -stimulates fibroblasts to synthesize collagen and elastin, protects collagen against MMP-induced degradation -takes part in angiogenesis in the dermis -influences skin thickness -influences melanin degradation and skin color -protects the skin against UV radiation -reduces sebum and limits the development of *Propionibacterium acnes*	-abnormal keratinization of the epidermis -excessive epidermal exfoliation -excessive skin dryness -reduced sebum secretion -follicular keratosis	[27,34,35,143,144,145]
Vitamin C (ascorbic acid)	citrus fruits, seaberry, dog-rose, blackcurrant, strawberry, raspberry, kiwi, hawthorn, rowan, cruciferous vegetables (Brussels sprouts, kohlrabi, broccoli, cabbage, cauliflower), spinach, chicory, green bean, red pepper, chives, parsley, nettle, five-flavor berry, oregano	-takes part in differentiation of keratinocytes -stimulates ceramide synthesis -influences skin hydration -takes part in collagen biosynthesis and the formation of the extracellular matrix, increases synthesis of GAGs -inhibits melanogenesis -protects against UV radiation, photoaging	-skin fragility -impaired collagen biosynthesis -thickening of the stratum corneum -impaired wound healing -subcutaneous bleeding -scurvy	[48,49,50,58,62]
Vitamin E (tocopherol)	wheat germ oil, sunflower oil, safflower oil, soybean oil, maize oil, cottonseed oil, palm oil, cereal products, nuts	-takes part in biosynthesis of collagen, elastin and GAGs -protects lipid structures of the stratum corneum -prevents the development of UV-induced erythema -has strong antioxidant properties	-exfoliative dermatitis -skin inflammation	[76,78,80,82,84,85,146]
Selenium (Se)	Brazil nuts, saltwater fish (yellowfin tuna, halibut, sardines), seafood, sea salt, meats, poultry, eggs, dairy products, cereals, cereal products, broccoli, white cabbage, asparagus, kohlrabi, garlic, onion, legumes, mushrooms	-acts as an antioxidant -is a cofactor for GPx, which removes harmful peroxides -reduces ROS-mediated inflammation and DNA damage -protects the skin from UV-induced oxidative stress -plays roles in fighting ageing and preventing ageing-related diseases	-several skin diseases, including psoriasis, acne vulgaris, and atopic dermatitis	[26,88,89,90,106,147]
Zinc (Zn)	red meat, fish, poultry, seafood (shrimp, oysters), nuts, pumpkin seeds, sunflower seeds, legumes, whole grains, dairy products	-prevents UV-induced skin damage -protects against photodamage -plays role in skin morphogenesis, repair and maintenance -modulates activity of 5α-reductase	-acrodermatitis enteropathica -atopic dermatitis, epidermolysis bullosa	[26,97,98,106,107,109,148,149]
Copper (Cu)	liver, seafood, nuts, oysters, seeds, some whole grains and legumes, chocolate, cocoa	-exhibits antimicrobial activity -stimulates collagen maturation -modulates melanin synthesis	-Menkes kinky hair disease (hypopigmented skin and hair, sparse, short, brittle scalp hair, pale, mottled, doughy skin)	[118,126,128,150]
Silicon (Si)	grains (rice, barley, oat, wheat), grain products (breakfast cereals, bread, pasta), root vegetables (carrots, beetroot, radish, onion, potatoes), beans, maize, fruit (bananas), dried fruit (raisins), nuts, field horsetail	-plays role in synthesis of collagen, elastin and GAGs -improves skin surface and its mechanical properties -improves keratin structure -increases resistance and thickness of nails and hair	-faster appearance of wrinkles -soft and brittle nails -slower wound healing	[135,137,138]

**Table 2:** Selected vegetable oils that are sources of fatty acids of importance for the skin.

Oil Example	Source	Fatty Acids Composition	Reference
Dry, sensitive and atopic skin			
Borage oil	*Borago officinalis*	γ-linolenic (26–38%), linoleic (35–38%), oleic (16–20%), palmitic (10–11%), stearic (3.5–4.5%), eicosenoic (3.5–5.5%), erucic (1.5–3.5%) acid	[163]
Evening primrose oil	*Oenothera biennis*	linoleic (70–75%), γ-linolenic (8–14%) acid	[161,164]
Blackcurrant seed oil	*Ribes nigrum*	linoleic (>40%), linolenic (10–20%), γ-linolenic (13–15%), stearidonic (2–3%) acid	[162,165]
Hemp oil	*Canabis sativa*	α-linolenic (16–19%), linoleic (55–58%), oleic (11–13%), γ-linolenic (4%), palmitic (5–6%), stearic (2.3–2.5%) acid	[166,167]
**Oily, combination and acne skin**			
Baobab oil	*Adansonia digitata*	linoleic (36%), palmitic (28.8%), oleic (25.1%) acid	[168,169,170]
Neem oil	*Azadirachta indica*	oleic (40%), stearic (16%), palmitic (14%), linoleic (21%) acid	[171,172]
Black cumin oil	*Nigella sativa*	γ-linolenic (50–60%), oleic (20%), eicosadienoic (3%), dihomo-γ-linolenic (10%), palmitic and stearic (˃30%) acid	[173,174]
Coconut oil	*Cocos nucifera*	capric (7%), lauric (49%), myristic (18%), palmitic (9%), oleic (6%), stearic (2%), linoleic (2%) acid	[175]
**Mature skin, photoaging, UV protection**			
Sea buckthorn oil	*Hippophaë rhamnoides*	linoleic (34–40%), α-linolenic (23–36%), palmitic (26.3%), oleic (15–20%) acid	[158,176,177]
Green coffee oil	*Coffea* *arabica*	linoleic (44%), palmitic (34%), oleic (9%), stearic (7%), arachidonic (3%), linolenic (1.5%) acid	[178,179]
Wheat germ oil	*Triticum aestivum*	linoleic (58.6%), α-linolenic (9.6%), oleic (11.2%), palmitic (17.9%), stearic (1.2%), palmitoleic (0.2%) acid	[180]
Barbary fig oil	*Opuntia ficus-indica*	linoleic (62–49%), palmitic (10.6–12.8%), oleic (13–23.5%), stearic (3.3–5.4%) acid	[181]
Argan oil	*Argania spinosa*	oleic (43–49%), linoleic (29–36%), palmitic (11–15%), stearic (4–7%) acid	[182,183,184]

**Table 3:** Classification of carotenoids, and their sources, functions and importance in skin health [219,220,221,222,223,224,225,226,227,228].

Classification	Example	Source	Function
Carotenes—the hydrocarbon carotenoids	β-carotene	beet root, apricots, cantaloupe, carrots, pumpkin, sweet potato, pink grapefruit, tomatoes, watermelon, mango, papaya, peaches, prunes, oranges, bilberry green fruits and vegetables such as green beans, broccoli, brussels sprouts, cabbage, kale, kiwi, lettuce, peas, spinach, acerola, nuts, oil palm, buriti (*Mauritia vinifera*), sea buckthorn (*Hippophaë rhamnoides*), camu-camu (*Myrciaria dubia*), rose hip (*Rosa canina*), alga *Dunaliella salina*	-conversion to retinoids (provitamin A carotenoids: α- and β-carotene, β-cryptoxanthin) -modulation of the enzymatic activity of lipoxygenases -enhancement of immune system function -antioxidant activity (ability to quench singlet oxygen, free radical scavenging (including superoxide anions and hydroxyl radicals) -reduction of lipid peroxidation in human skin -promotion of secretion of transcription factors and growth factors -suppression of UVA-mediated induction of MMP-1, MMP-3, and MMP-10 -protection of dermal collagen against UV-induced oxidation of proline -promotion of synthesis of procollagen type I -anti-inflammatory properties -protection of cell membranes and tissues against effects of UV light -protection of skin against UVB-induced photoaging and photocarcinogenesis -reduction of sensitivity to UV radiation-induced erythema -protection from sunburn -skin-lightening effects -improvement of skin condition (hydration, skin texture, radiance, elasticity, reduction in furrows and wrinkles)
	lycopene	tomato and its derivatives, such as juices, soups, sauces, and ketchup, cherry, guava, watermelon, papaya, peaches, grapefruit, asparagus, rose hip	
Xanthophylls—the oxygenated carotenoids	lutein	green and dark green leafy vegetables, like broccoli, brussels sprouts, spinach, parsley, pumpkin, acerola, rose hip, *Tropaeolum majus*, caja (*Spondias lutea*), camu-camu (*Myrciaria dubia*), microalgae *Chlorella vulgaris*, eggs	
	zeaxanthin	broccoli, brussels sprouts, spinach, parsley, maize, pequi (*Caryocar villosum*), microalgae *Chlorella saccharophila*	
	astaxanthin	aquatic animals, such as lobster, crab, salmon, trout, krill, shrimp, crayfish and crustacea, microalgae *Phaffia rhodozyma*, *Chlorella vulgaris*, Haematococcus pluvialis, yeast	
	antheraxanthin	many plants, especially maize	
	α-, β-cryptoxanthin	many colored plants as maize and papaya, sea buckthorn	
	capsanthin	peppers, paprika	

## Data Availability

Data sharing not applicable.
